# Qualitative Perspectives of Emergency Nurses on Electronic Health Record Behavioral Flags to Promote Workplace Safety

**DOI:** 10.1001/jamanetworkopen.2023.9057

**Published:** 2023-04-20

**Authors:** Emily F. Seeburger, Rachel Gonzales, Eugenia C. South, Ari B. Friedman, Anish K. Agarwal

**Affiliations:** 1 Department of Emergency Medicine, Perelman School of Medicine, University of Pennsylvania, Philadelphia; 2 Center for Emergency Care Policy and Research, University of Pennsylvania, Philadelphia; 3 Penn Urban Health Lab, University of Pennsylvania, Philadelphia; 4 Penn Medicine Center for Health Care Innovation, University of Pennsylvania, Philadelphia

## Abstract

**Importance:**

Emergency nurses experience high levels of workplace violence during patient interactions. Little is known about the efficacy of behavioral flags, which are notifications embedded within electronic health records (EHRs) as a tool to promote clinician safety.

**Objective:**

To explore the perspectives of emergency nurses on EHR behavioral flags, workplace safety, and patient care.

**Design, Setting, and Participants:**

In this qualitative study, semistructured interviews were conducted with emergency nurses at an academic, urban emergency department (ED) between February 8 and March 25, 2022. Interviews were audio recorded, transcribed, and analyzed using thematic analysis. Data analysis was performed from April 2 to 13, 2022.

**Main Outcomes and Measures:**

Themes and subthemes of nursing perspectives on EHR behavioral flags were identified.

**Results:**

This study included 25 registered emergency nurses at a large academic health system, with a mean (SD) tenure of 5 (6) years in the ED. Their mean (SD) age was 33 (7) years; 19 were women (76%) and 6 were men (24%). Participants self-reported their race as Asian (3 [12%]), Black (3 [12%]), White (15 [60%]), or multiple races (2 [8%]); 3 participants (12%) self-reported their ethnicity as Hispanic or Latinx. Five themes (with subthemes) were identified: (1) benefits of flags (useful advisory; prevents violence; engenders compassion), (2) issues with flags (administrative and process issues; unhelpful; unenforceable; bias; outdated), (3) patient transparency (patient accountability; damages patient-clinician relationship), (4) system improvements (process; built environment; human resources; zero-tolerance policies), and (5) difficulties of working in the ED (harassment and abuse; unmet mental health needs of patients; COVID-19–related strain and burnout).

**Conclusions and Relevance:**

In this qualitative study, nursing perspectives on the utility and importance of EHR behavioral flags varied. For many, flags served as an important forewarning to approach patient interactions with more caution or use safety skills. However, nurses were skeptical of the ability of flags to prevent violence from occurring and noted concern for the unintended consequences of introducing bias into patient care. These findings suggest that changes to the deployment and utilization of flags, in concert with other safety interventions, are needed to create a safer work environment and mitigate bias.

## Key Points

**Question:**

How do emergency nurses view electronic health record–based behavioral flag notifications in relation to workplace safety and patient care?

**Findings:**

This qualitative study interviewed 25 emergency nurses and described their views on workplace violence, safety, and patient care. Electronic health record flags were described as providing a helpful advisory for nurses but were thought to be unenforceable and riddled with process roadblocks as well as potentially perpetuating bias in care.

**Meaning:**

These findings suggest opportunities for improvements in the implementation of behavioral flags as well as system-level approaches to addressing the risk of workplace violence in health care settings.

## Teaser

This qualitative study examines perspectives of emergency nurses on behavioral flags in electronic health records in relation to workplace safety and patient care.

## Introduction

Workplace violence from patients to clinicians is unfortunately common in emergency care, as 70% of emergency nurses and 20% of physicians report being physically assaulted and an even greater percentage report being verbally harassed by patients.^1,2^ More alarming is that 46% of nurses reported experiencing workplace violence or harassment during their most recent shifts.^3^ Of these interactions, one-third were physical assaults.^3^ Health care clinician exposure to workplace violence has been linked to absenteeism, lowered morale, and decreased productivity.^4,5,6^ Research has demonstrated that burnout and issues of workplace safety can lead to more medical errors, which can be especially dangerous in emergency care settings.^7,8^

Research has identified risk factors associated with hospital-based violence. These risk factors range from societal factors (outside conflict), institutional factors (long wait times, emergency department [ED] crowding, fallout from death notifications or other difficult conversations, barriers to reporting violent incidents), and patient characteristics (dementia, delirium, intoxication, or undertreated mental illness).^9,10,11^ There is an absence of data on which health system–based interventions are effective for reducing risk of workplace violence.

A handful of solutions designed to prevent or mitigate patient to clinician violence exist. A US survey found that 40% of EDs screen for weapons, 38% have metal detectors, 16% provide staff with violence prevention workshops, and 10% offer self-defense training.^1^ Electronic health records (EHRs) offer a way to address clinical workplace violence—primarily through behavioral notification or alerts commonly referred to as flags. These flags are created after an initial incident of verbal harassment, physical assault, or another behavioral issue to alert and notify future clinicians. The flag is then embedded within the patient’s health record, and subsequent clinicians are notified via an immediate alert (eg, “pop-up”) requiring formal acknowledgment. These flags are intended to act as an early notification for clinicians to be aware of safety, violence, or behavioral concerns when caring for patients in future encounters.

While behavioral flags represent a potential solution to addressing workplace violence in the ED, little is known about their utility, effectiveness, or potential for unintended consequences on care. The EHR provides a unique landscape at the intersection of patient experience, clinical care, and equity. A recent study found that Black patients were 2.5 times more likely to have negative descriptor language in the EHR.^12^ Additionally, hospital-based physicians report that the EHR negatively impacts clinician-patient communication.^13^ Behavioral flags may compound these existing issues. Less is known about the perceptions and impact of behavioral flags in a clinical environment.^11^ This study explores this issue by going to the source closest to these flags: emergency nurses, who have the most interaction with patients and who are at the highest risk of violence. The objective of this qualitative study was to assess emergency nurse perceptions, beliefs, and attitudes toward EHR behavioral flags.

## Methods

### Study Design and Participants

This qualitative study used semistructured interviews of registered emergency nurses at an urban, academic, level I trauma center ED from February 8 and March 25, 2022. Participants were consented verbally. Interviews were designed to focus on their perceptions, beliefs, and attitudes toward the use of EHR behavioral flags. This project was approved by the University of Pennsylvania Institutional Review Board. Written consent was waived because the study presented no more than minimal risk to participants. The study followed the Consolidated Criteria for Reporting Qualitative Research (COREQ) reporting guideline.

Using a convenience, snowball sampling technique, participants were recruited in person or via email across weekday and evening shifts. For variation, sampling was conducted across different shifts, times of day, and points in the shift. The sample size allowed for thematic saturation. Participants were compensated with a $30 gift card for their time.

### Behavioral Flag Process

Within the study site health system, any ED clinician (eg, physician, nurse, resident physician, or technician) can place a behavioral flag in the EHR after an incident of verbal, physical, or sexual assault or another safety issue (eg, a patient attempting to break into a medication cart). The submission includes information about the time and date of the incident, the patient, and an open-text field to describe the incident. Variation exists at the hospital site level on if, and when, flags are reviewed by an administrator or leader. At the study site, flags can be reviewed by the ED unit-based multidisciplinary leadership team. The flag is displayed as both an icon in the EHR and as a pop-up notification when the patient medical record is opened. There are no specific policies or procedures that dictate actions that must be taken by staff if a flag is present; instead, the behavioral flag system was created to make clinicians aware of a patient who may compromise their safety. Furthermore, there is no standardized process for the removal of a flag. In the study health system, fewer than 1% of all ED patients have a behavioral flag.

### Study Procedure

Semistructured interviews with participants were conducted in person or via telephone by a trained qualitative research team member (E.F.S.). The interviewer used an interview guide developed by all members of the study team, consisting of qualitative researchers and emergency physicians with expertise in burnout and equity (E.F.S., E.C.S., A.B.F., and A.K.A.) (eAppendix in Supplement 1), and pilot tested (E.F.S. and R.G.). The guide included open-ended questions and subsequent probing questions about the usefulness and impact of flags. Participants self-reported demographic characteristics such as their age, sex, race (Asian, Black, White, or multiple races) and ethnicity (Hispanic or non-Hispanic [Latinx or non-Latinx]), and ED tenure. Informed by research indicating racial disparities in behavioral flag issuance, nurses were asked about their perspectives on how racial bias may be introduced and its impact on flagging patient behavior. Recently published data note disparities in flag prevalence, with Black patients flagged at higher rates than White patients (4.0 vs 2.4 flags per 1000 patients) and male patients and those who utilize Medicaid also flagged more often.^14^ Thus, the decision to include this focus question within these interviews was made a priori.^14^ Interviewers noted that the number of flags tended to be greater for Black patients, disproportional to their representation in the patient population. Interviewers followed up with probing questions asking the participant why they did or did not believe racial bias plays a role. All interviews were recorded, transcribed, and deidentified using the Rev professional transcription service^15^ and reviewed for accuracy.

### Thematic Analysis

A thematic analysis of interview responses was conducted using the constructivist paradigm. An initial codebook using a grounded theory approach for analyzing interview responses was developed, then revised iteratively throughout the coding process. Revisions were applied to future codes as well as retroactively to already-coded segments wherein the new code was a more appropriate categorization, known as a constant comparative coding approach. Two authors (E.F.S. and R.G.) assessed a subset of 5 transcripts (20%) for interrater reliability and reached consensus. Member checking was not conducted. NVivo, version 12 (QSR International), was used for data storage, management, and coding.^16^ Data analysis was performed from April 2 to 13, 2022.

## Results

This qualitative study included 25 registered emergency nurses at a large academic health system, with a mean (SD) tenure of 5 (6) years in the ED. Participant characteristics are summarized in Table 1. Their mean (SD) age was 33 (7) years; 19 were women (76%) and 6 were men (24%). Participants self-reported their race as Asian (3 [12%]), Black (3 [12%]), White (15 [60%]), or multiple races (2 [8%]); 2 participants (8%) did not report this information. Participants self-reported their ethnicity as Hispanic or Latinx (3 [12%]) or non-Hispanic or non-Latinx (22 [88%]). The demographic background of study participants mirrored that of nursing staff throughout the department.

**Table 1.** Self-reported Characteristics of Emergency Nurse Perspectives on Behavioral Health Flags

Characteristic	Respondents (N = 25)^a^
Age, mean (SD) [IQR], y	33 (7) [25-51]
Sex	
Female	19 (76)
Male	6 (24)
Race	
Asian	3 (12)
Black	3 (12)
White	15 (60)
Multiple races^b^	2 (8)
Not reported	2 (8)
Ethnicity	
Hispanic or Latinx	3 (12)
Non-Hispanic or non-Latinx	22 (88)
Emergency department tenure, mean (SD) [IQR]	5 y (6 y) [1 mo to 28 y]

^a^ Unless indicated otherwise, values are expressed as No. (%) of respondents.

^b^ These participants identified as biracial but only inclusive of the racial identities listed (ie, Asian, Black, or White).

Five main themes were identified using directed content analysis. *Benefits of flags* (theme 1) was used when positive use or application of flags was discussed. Content was categorized as *issues with flags* (theme 2) when a negative use, application, or downstream effect of behavioral flags was discussed. *Patient transparency* (theme 3) was applied when opinions related to greater information sharing with patients about behavioral flags in their EHR were shared. Content was categorized as *system improvements* (theme 4) when suggestions or ideas to improve the behavioral flags process or increase ED workplace safety were provided. Finally, content was categorized as *difficulties of working in the ED* (theme 5) when narratives illuminated the challenges of working in the ED contributing to behavioral flag use were shared. The 5 themes, subthemes, and illustrative quotes are summarized in Table 2.

**Table 2.** Themes, Subthemes, and Illustrative Quotes in a Qualitative Study of Emergency Nurse Perspectives on Behavioral Health Flags

Theme and subtheme	Illustrative quote (participant)
**Benefits of flags**	
Useful advisory	“I definitely take notice of them and I do read them.… It makes me a little bit more, um, just aware in terms of, okay, I just need to be aware of this. And if that patient does respond this way, just back myself out for safety reasons.” (Participant 9)
Prevents violence	“So I recently actually just had a patient who came in complaining of homicidal ideations and some of the flags were actually really beneficial ‘cause it talked about him carrying a weapon, concealing a weapon and all this other stuff. So I was able to, ‘cause like most of the time, if we have homicidal patients or suicidal patients for one, the patient care tech will sit on it and then go through their belongings.… And the patient was like, he literally told me, they took my knife away. But if I had my knife, I would cut anybody that’s closest to me.” (Participant 2)
Engenders compassion	“The patient population has been very different for me. I think it’s just because of the community and like the obstacles they face. It has been almost like a culture shock for me.… I think [the flag] helps me be like more compassionate and also just go in there with more sensitivity towards them.” (Participant 10)
	“I think I’m almost nicer to them when I go into the room. Like I had someone last week, all of her interactions were flags from within like the past month. So I try to almost be nicer to them to gauge where they are initially, because you just don’t know if they’re already in a bad mood, if they were in a bad mood from the waiting room, especially when we have long waits, if that’s making it worse.” (Participant 4)
**Issues with flags**	
Administrative and process issues	“Sometimes, you know, I don’t have time to sit and fill paperwork. I don’t have time sometimes to just sit down there and write something down. I’m going to the next patient. And then whenever I’m done, it’s 12 hours. I just wanna get the heck out, honestly. Yeah. So things end up not even getting reported a lot of the times with me.” (Participant 11)
	“I filled out safety nets about aggressive patients. And I’ve never heard anything else again. Yeah. I like literally don’t know whatever happens to it again. Or, I mean, I get like a little thing of your safety net has been evaluated.… Why, why do I wanna bother writing the safety net then?” (Participant 3)
Unhelpful	“I don’t like the way they pop up. I personally don’t think they’re like user friendly. It’s a yellow window. It’s small writing. It doesn’t immediately say what they’re flagged for.” (Participant 8)
Unenforceable	“I don’t think the redbook means anything cause I’ve seen multiple patients that are here admitted, you know, getting treatment.… We have to take care of them. People come in that are like, that are redbooked that have like a cut. Yeah. And we still take them back. So it’s completely annoying.” (Participant 7)
Bias	“But maybe like nursing care or like tech care, I feel sometimes they might treat them a little bit differently if they’ve had a flag in the past. Even if it’s like anything from like stealing things to hurting staff or just being loud. I think people do treat them differently, unfortunately.” (Participant 1)
	“Do I think it’s intentional? I don’t think so, but I think this just happens because the population that we have here is mostly African American. And most of them, I would say the ones that usually are flagged, the ones that come in for like, who are high on PCP or drugs, or, can be verbally abusive, you know.… I feel that’s primarily why it [being flagged] happens.” (Participant 6)
	“I think there may be an underlying bias that people don’t even necessarily realize. I would say a majority of our staff is white and I just feel like people are a little intimidated by people that aren’t necessarily from the same background, same skin of color. I don’t think people intend to necessarily have a bias, but like I said, I sort of think it’s more of a subconscious type deal.” (Participant 14)
	“I feel like sometimes different attitudes with different nursing staff, [the patient’s attitude] will come across a different way. So whether the nurse was more aggressive or less tolerant of the behavior then maybe things escalated instead of trying to figure out the reason. And I’m not saying a lot of people don’t have reasons to put flags in, but I think sometimes I’m more tolerant. I try to understand what’s happening.” (Participant 21)
Outdated or uncharacteristic	“I also don’t like really dated flags either. 2015, and then this person’s been here for years and there’s nothing, I’ve never had a problem, but I don’t even know what the scenario was. Did somebody antagonize the person and they’re just defending themselves? You know? So, we’re all human, I’m tired. Sometimes I’m on my third, 12 [hour shift]. You talked to me some way. I might be ready to snap at you. I’m a human being. So whatever, you know, we all lose it.” (Participant 3)
**Patient transparency**	
Patient accountability	“For me, I think they should know that we’ve made other people aware, like include that security and whoever it is that’s supervising is aware of your behavior on such and such day.” (Participant 4)
Damages patient-clinician relationship	“So there’s such a nonstandardized way of them being placed currently. As we said, there can be kind of irrelevant reasons why they’re placed and it can be very damaging to, I think, a patient’s relationship with their providers and with [the health system] as a whole, if they’re notified.” (Participant 11)
**System improvements**	
Process	“Maybe they could, over time, eliminate it. I’ve read plenty of flags from the early 20 teens and it’s like, okay, that was six, seven years ago. I’ve had a great time with this person. They’ve been nothing but calm and fine. But then everywhere they go, the first thing that pops up is gonna be this big thing. And usually you don’t read any of it. You’re just like, oh, well this person’s aggressive. So, it could’ve happened yesterday as opposed to, you know, nine years ago. And then maybe they were a drug addict. Maybe they were going through a terrible time in their life. Maybe there weren’t people listening to them.” (Participant 3)
Built environment	“It’s [the triage room] like a four by six room. Yep. With a computer desk in it. There’s not enough space. What they teach in all those behavioral health classes don’t work. They teach you stay an arms length away from the patient. That’s what they teach us. We say protect us. And they say, take this class. Well, we can’t even execute what’s taught to us in the class because it’s impossible to be at arm’s length away from the patient in that room.… And also there’s really no safe egress out of there at all.” (Participant 5)
Human resources	“I think the MOAB [training] is a joke and everybody treats it as a joke ‘cause it is a joke. It’s that clinical cold way of dealing with violence and it never fits that algorithm in a real life situation.” (Participant 13)
	“There’s also that respect piece and feeling valued. So when you’re beaten up at work and you complain and no one does anything, how do you think that you feel valued? You feel like sh-t. Feel like absolute sh-t.” (Participant 5)
**Difficulties of working in the ED**	
Harassment and abuse	“This guy is threatening to wait for me outside. And then in quotes, light me up and is like verbally abusing me and threatening me with an actual gun. It wouldn’t be that hard for somebody to just wait, wait around until like seven, eight o’clock and they see me come out.… So like, what if they follow me to my car? Like I’ve been followed from work before by a patient. I mean nothing happened but we get all kinds of things. I mean, this person, you know, was just, I think they were a little messed up in the head.” (Participant 11)
	“[A patient] then was joking with a med student, told the med student I was his girlfriend. Later, told me that he liked my perfume. It just continued and continued and continued. I felt uncomfortable, but I felt as though it was an accident and then he just continued to be creepy.” (Participant 8)
Unmet mental health needs of patients	“We need someone here that can help with that [psychiatric care]. That’s their profession. Yeah. That our docs are great and our nurses are adequate enough to like provide, but we don’t know the ins out of all that stuff. That’s not our expertise.” (Participant 13)
COVID-19–related strain and burnout	“With COVID, I think a lot of healthcare professionals have tried to just have a conversation of why aren’t you vaccinated, you should get the vaccine. And that has also just turned into an argument between people with a lack of education and resources and everything. And it’s just also just escalating more problems and issues.” (Participant 2)

Abbreviations: ED, emergency department; MOAB, management of aggressive behavior; PCP, phencyclidine.

### Theme 1: Benefits of Flags

#### Useful Advisory

Nurses identified strengths of behavioral flags, particularly that they provided a useful advisory that a patient may be verbally or physically aggressive. Similarly, participants who had placed a flag in a patient’s EHR did so to hopefully provide awareness to future clinicians.

#### Prevents Violence

In extreme cases, more than half of ED nurses believed that the behavioral flag prevented a potentially dangerous interaction, as the flag enabled the nurse to be more prepared and activate other safety training in advance. This is particularly important for patients with a flag documenting past homicidal ideation, as this forewarning prompts nurses to search for concealed weapons that were not captured on initial screening. Relatedly, behavioral flags for some patients with a violent history allowed staff to strategically place these individuals in clinical areas with additional staff support in the event that a violent or aggressive action occurred.

#### Engenders Compassion

Approximately one-fifth of nurses indicated that the presence of a behavioral flag made them more understanding or compassionate toward a patient, particularly if the patient was thought to be unhoused. Almost all participants described underlying, unmet mental health and substance use needs of patients that commonly contribute to behaviors leading to a flag. This was then frequently followed up by nursing desires to provide more behavioral health services from the ED.

### Theme 2: Issues With Flags

#### Administrative and Process Issues

Several issues with the documentation process for behavioral flags were identified. Numerous administrative, or process, concerns were outlined. In particular, nurses expressed concern that the process to place a flag in the EHR is too long, is complex, and requires information that is tangential to the goal of the flag (eg, race, ethnicity, or sex) and, once submitted, there is little to no follow-up from supervisors or the health system. These issues often make nurses ask, “Why bother?”—especially at the end of a long shift.

#### Unhelpful

The majority of participants viewed flags as unhelpful, noting that actual patient discipline “never happens” or the presence of a flag “doesn’t change anything.” These participants indicated that while a flag may make them proceed with increased caution, the flag itself will not prevent a patient from becoming violent. Complaints over the annoyance of another EHR-based, pop-up window or the formatting of the notification (eg, small text, difficult to read) were also discussed.

#### Unenforceable

In extreme situations and in a minority of cases in which a patient receives multiple flags for repeated violence or egregious behavior, participants cited concerns regarding the overall effectiveness of the flag notification. Nearly all nurses described the repeated flags as having “no teeth” and being largely unenforceable because of the Emergency Medical Treatment and Labor Act.

#### Bias

There was concern that the flag may negatively bias the patient-nurse interaction. Concerns over the introduction of bias were either theoretical or had been witnessed, or the participant had admitted to shifting their approach and treating the patient “differently” in a way they viewed as negative, whether implicitly or explicitly. More than half of participants said that flags prompted them to approach the patient with caution informed by the flag documentation. Participants were insistent that a change in approach, however, did not impact the quality of clinical care provided.

While participants acknowledged that flag placement can be biased against individuals who use the ED frequently or people with addiction, many felt that there was no association between the racial or ethnic identity of the patient and whether they received a flag. The notion that more Black than White ED patients have flags was largely attributed to the patient population. Others, however, did feel that race and ethnicity play a role in who receives a flag, what kinds of behaviors are tolerated, and to what degree. For example, several described a greater tolerance for harassment and abuse from White patients by physicians, making them less likely to intervene or provide support to nurses.

#### Outdated or Uncharacteristic

There was concern over inappropriate or irrelevant flags. All participants discussed the subjective nature of the flag narrative content. This content varied between verbal harassment and sexual assault of staff without regulation or standardization.

Participants also noted that on some occasions, staff may escalate situations with patients, which can cause an unnecessary issuance of a flag. The majority of participants noted that burnout and staffing issues have contributed to stress and place nurses on edge, making them more likely to “snap.” A few participants also mentioned that some of their colleagues generally have more of a propensity to be confrontational than others.

Participants also acknowledged that wait times in the ED are long, that patients may be in pain or otherwise feeling unwell, and that the circumstances surrounding the ED visit may provoke patient behavior that is otherwise uncharacteristic. Relatedly, nurses indicated a problem with patients having flags from years prior with no subsequent issues, and therefore, the flag is irreflective of the individuals’ actual demeanor.

### Theme 3: Patient Transparency

#### Patient Accountability

Nurses interviewed were asked whether patients should always know if their EHR contains a behavioral flag. Nurses described the need for patient accountability, and they stated that notifying them would make patients “aware” that security and supervising staff knows of their behavior.

#### Damages Patient-Clinician Relationship

There was concern that more widespread notification of a behavioral flag would be “damaging” to future patient-clinician relationships and the patient’s overall relationship with the health system.

### Theme 4: System Improvements

#### Process

In response to the myriad flag issues outlined, nurses described potential improvements to the process. The following improvements were mentioned: make the reporting form easier to complete, increase follow-up and communication after a flag is submitted, standardize the issuing of a flag, provide greater education for staff on the workplace violence reporting process, and perform a continual review for flag removal.

#### Built Environment

Improvements to the physical environment of the ED were also suggested. Participants overwhelmingly described the danger presented by the physical layout of the triage room as well as issues with patient room doors that can only open in 1 direction. The desire for increased video surveillance and more security staff (especially for more secluded areas), as well as more careful screening of visitors, was also discussed.

#### Human Resources

Human resource solutions were also mentioned. Solutions included the following: provide more effective and/or role-playing de-escalation trainings, make a more concerted effort to hire nursing staff from diverse backgrounds, and hire psychiatrists specifically for the ED to more readily address the mental health and substance use difficulties overrepresented in patients with behavioral flags and the high-utilizer population of the ED overall.

#### Zero-Tolerance Policies

Finally, there was a desire for lower tolerance of incidents of verbal and physical violence from ED and health system leadership, especially when an extreme incident of workplace violence occurs. This included more forceful “no tolerance” policies in the case of harassment or violence, greater legal support, and acknowledgment that this level of harassment and abuse sustained daily is a serious issue.

### Theme 5: Difficulties of Working in the ED

#### Harassment and Abuse

When asked if they had ever documented an incident to issue a behavioral flag, nurses often described the incident that prompted it. These experiences were aggregated, describing a breadth of daily violence and harassment. The experiences described included sexual assault, physical violence, and daily verbal harassment.

#### Unmet Mental Health Needs of Patients

In many instances, participants attributed the inappropriate patient behaviors to underlying and unmet mental health needs and substance use disorder needs. They stated that greater resources and access to care are needed.

#### COVID-19–Related Strain and Burnout

More than half of participants also noted that the COVID-19 pandemic contributed to more tense interactions. They attributed this change to conversations about vaccination, masking, or ripple effects related to staffing, long wait times, health system strain, and increases in gun violence.

## Discussion

This qualitative study of emergency nurse perceptions of EHR behavioral flags aimed to address a critical gap in understanding these flags and their potential for reducing workplace violence from patients to staff and implications for patient care. This study had 3 important findings.

First, while flags provided staff with a useful warning, fulfilling in part the initial motivation for their use, several concerns emerged that nurses viewed as precluding flags from truly moving the needle in promoting clinician safety. These seemingly contradictory viewpoints present a nuanced view of both useful and nonuseful components of flags. From these participant perspectives, there is a greater need for consistency in the system-level response to a flagged incident, particularly by those reviewing the documentation of an initial incident, and a consistent response to patients who display the largest risk toward staff safety is also needed. From these perspectives of ED nurses, the lack of leadership follow-up, a belief that the flags do not decrease violence, and complaints of a long and complex documentation system create a dangerous, self-enforcing mechanism in which workplace violence and harassment is underreported and thus leaves all staff, patients, and visitors at risk.

Second, the disparate perspectives on the influence of bias in flags provided interesting insight into their potential unintended consequences. Emerging research has reported observable implicit bias in EHR descriptions of patients, in the perception of how violent a patient is, and in clinical decision-making.^12,17,18^ Despite the nurses’ mixed perspectives on the intersection of race and flagging in this study, other work by this team has shown that Black patients disproportionately receive flags compared with White patients.^14^ As part of the expressed desire for better and more effective trainings to address workplace violence, these findings suggest that it would be worthwhile to include trainings that specifically address the relationship between racism and flags as well. Other procedures, such as a periodic review of patient behavioral flags and the utilization of a patient advisory board to provide policy guidance, could be introduced in an effort to mitigate systemic racism related to flags. Addressing issues of health inequity is also fundamental to improving public health.

Finally, participants readily identified solutions to remediate the behavioral flag and workplace safety issues described in the interviews. The need to reconfigure triage was something mentioned in almost every interview, as its layout posed major staff and patient safety risks. Other built environment solutions, like bidirectional opening doors to ensure staff always have safe passage out of patient rooms, were also repeatedly mentioned. Other suggestions provided, especially those to improve documentation and communication, could also be implemented to remedy process complaints. These findings suggest that the issue of violence in the ED can be readily improved.

### Limitations

This qualitative study has several limitations. Participants were registered emergency nurses from an urban, academic health system. The sample comprised mainly White women, reducing data variance and insight from individuals in other roles and those with diverse racial and ethnic backgrounds. However, our sampling approach allowed for more in-depth understanding of nurse relationships with behavioral flags, which is especially relevant given that nurses are the primary clinicians interacting with patients in the ED; typically, there is 1 registered nurse for every 4 to 5 patient rooms. Nurses, compared with other ED staff, are also less likely to feel safe.^19^ Participants volunteered and thus sampling bias was present. Lastly, the increased stress and workplace violence–related events attributable to COVID-19 and gun violence in this setting at the time of this study may have influenced responses. However, the increased focus on improving mental health and employee wellness also as a result of those 2 factors makes this work especially timely and important.

## Conclusions

Emergency nurse perspectives on the effectiveness of behavioral flags vary. For many, flags serve as a helpful alert to approach patient interactions with caution or employ safety skills learned through other trainings. The ability of flags to prevent violence from occurring altogether seems unlikely. The findings of this study suggest that changes are needed to improve the process from initial documentation through follow-up and enforcement, especially for patients who pose a high risk of violence. Changes to the clinical environment are also needed. It is important to underscore that there is risk of bias, particularly racial bias, in the issuing of flags and clinician reactions to reading them in a patient’s EHR, and an effort to implement measures to mitigate bias while keeping nurses and staff safe should be considered. One kind of intervention alone is not enough to prevent violence in the ED altogether; future research should explore what other procedures or policies, in concert with behavioral flags, are most effective in reducing incidents of harassment or assault in the ED. Future work by this research team will explore the outcomes associated with flags on patient care, particularly whether having a flag in a patient’s EHR in perpetuity affects their care.

## Notes

Supplement 1. **eAppendix.** Clinician Interview Script

Supplement 2.
Data Sharing Statement
