# Supplementary material for: Qualitative Perspectives of Emergency Nurses on Electronic Health Record Behavioral Flags to Promote Workplace Safety
Source: JAMA Netw Open. 2023 Apr 20;6(4):e239057. doi: 10.1001/jamanetworkopen.2023.9057 (PMC10119742; doi:10.1001/jamanetworkopen.2023.9057)
Supplement: Supplement 1. — eAppendix. Clinician Interview Script [file jamanetwopen-e239057-s001.pdf]

Supplementary Online Content

Seeburger EF, Gonzales R, South EC, Friedman AB, Agarwal AK. Qualitative perspectives of emergency nurses on electronic health record behavioral flags to promote workplace safety. *JAMA Netw Open*. 2023;6(4):e239057. doi:10.1001/jamanetworkopen.2023.9057

eAppendix. Clinician Interview Script

This supplementary material has been provided by the authors to give readers additional information about their work.

© 2023 Seeburger EF et al. *JAMA Network Open*.

eAppendix. Clinician Interview Script

INTRODUCTION

Hello, my name is ______. I am from the University of Pennsylvania and am working with a team of doctors who want to know more about the application and usefulness of behavioral flags (also called notifications) linked to the electronic health records as a mechanism to promote safety.

We know from prior research that the complex needs that present in the emergency department create a dynamic and challenging work environment for providers (physicians, resident trainees, nurses, advanced practice providers) and heighten the risk for workplace violence. Our goal is to use your feedback and the feedback of about 30 other providers who we are interviewing, to improve workplace safety in the ED while also ensuring care equity. We want you to be completely honest.

Before we start our interview it's important to go over a few things.

1. This interview will take about 20-30 minutes.
2. This interview is completely confidential. The answers you give here will not impact your current or future care/employment at Penn Medicine. You are free to share as much or as little about your experiences as you would like, and we can skip a question or stop the interview at any point.

© 2023 Seeburger EF et al. *JAMA Network Open*.

3. We will record and then transcribe the interview. All identifying information will be removed and we will destroy the recording. Your name and any identifying information will never be used in connection with the information from your survey or this interview.
4. Participation in this research is completely voluntary. You may opt out at any time if you wish.
5. You will receive a $30 gift card for your time.

PROVIDER QUESTIONS

First, we want to understand how you utilize and respond to behavioral flags in patient ED EHR.

1. Think back to a time where you interacted with a patient who was agitated or otherwise verbally or physically harmful. Did this patient have a behavioral flag already in their EHR?
   a. If yes, did that flag help you approach that patient interaction? How so?
   b. If no, would one have been helpful? In what way(s)?
   c. As a result of this interaction, did you create a behavioral flag in the patient's EHR? What influenced your decision to create or not create a flag?

2. Walk me through your reaction and thought process when you see a behavioral flag notification.

© 2023 Seeburger EF et al. *JAMA Network Open*.

a. Do you read through the notification? Always? Why or Why not?
b. Do you dismiss it without reading?
c. What content do you find helpful? Or not?
d. In your opinion, how does the flag impact you and the care you deliver?

Next, we want to gain your perspective on transparency and equity regarding the use of behavioral flags.

3. Patients are unaware if their EHR has a behavioral flag associated with it, unless they received a formal warning letter from the hospital. What do you think about this?
    a. Do you think all patients should be made aware if they have a behavioral flag? Why/why not?
    b. In what ways could greater transparency be helpful? Harmful?

4. How might issues of health equity emerge with the usage of behavioral flags?

5. In what ways have you altered your patient care, or witnessed another provider alter their care of a patient as a result of a behavioral flag? What was the impact on the patient?

Now we are very interested to hear your suggestions for how behavioral flags can be improved.

© 2023 Seeburger EF et al. *JAMA Network Open*.

1. What could be changed about how flags are created or displayed to make them more useful?
2. What could be changed about the flag creation process to make it more transparent to patients?
3. What else could the hospital do address issues of workplace safety in the ED?

Thank you for participating in this interview.

Brief Demographic Collection:

- Age
- Gender
- Race
- Ethnicity
- Years working in the ED

© 2023 Seeburger EF et al. *JAMA Network Open*.
