# Supplementary material for: Qualitative Perspectives of Emergency Nurses on Electronic Health Record Behavioral Flags to Promote Workplace Safety
Source: JAMA Netw Open. 2023 Apr 20;6(4):e239057. doi: 10.1001/jamanetworkopen.2023.9057 (PMC10119742; doi:10.1001/jamanetworkopen.2023.9057)
Supplement: Supplement 2. — Data Sharing Statement [file jamanetwopen-e239057-s002.pdf]

Data Sharing Statement

Seeburger. Qualitative Perspectives of Emergency Nurses on Electronic Health Record Behavioral Flags to Promote Workplace Safety. *JAMA Netw Open*. Published April 20, 2023. doi:10.1001/jamanetworkopen.2023.9057

Data
Data available: No
